# Secondary carnitine deficiency during refeeding in severely malnourished patients with eating disorders: a retrospective cohort study

**DOI:** 10.1186/s40337-024-01054-4

**Published:** 2024-07-09

**Authors:** Miho Imaeda, Satoshi Tanaka, Tomoko Oya-Ito, Mariko Uematsu, Hidetsugu Fujigaki, Kuniaki Saito, Masahiko Ando, Norio Ozaki

**Affiliations:** 1 Department of Clinical Oncology and Chemotherapy, Nagoya University Hospital, 65 Tsurumai, Showa, Nagoya, 466-8550 Aichi Japan; 2 Department of Psychiatry, Nagoya University Graduate School of Medicine, 65 Tsurumai, Showa, Nagoya, 466-8550 Aichi Japan; 3 NHO Higashiowari National Hospital, 1301, Omorikita 2, Moriyama, Nagoya, 463-0802 Aichi Japan; 4 NHO Nagoya Medical Center, 1-1, Sannomaru 4, Naka, Nagoya, 460-0001 Aichi Japan; 5 Department of Nutrition, Shubun University, 6 Nikkocho, Ichinomiya, 491-0938 Aichi Japan; 6 Department of Advanced Diagnostic System Development, Fujita Health University Graduate School of Health Sciences, 1-98 dengakugakubo, kutukakecho, Toyoake, 470-1192 Aichi Japan; 7 Center for Advanced Medicine and Clinical Research, Nagoya University Hospital, 65 Tsurumai, Showa, Nagoya, 466-8550 Aichi Japan; 8 Pathophysiology of Mental Disorders, Nagoya University Graduate School of Medicine, Institute for Glyco-core Research (iGCORE), Nagoya University, 65 Tsurumai, Showa, Nagoya, 466-8550 Aichi Japan

**Keywords:** Anorexia nervosa, Carnitine, Eating disorders, Fatty acid metabolism, Liver damage, Refeeding

## Abstract

**Background:**

Secondary carnitine deficiency in patients with anorexia nervosa has been rarely reported. This study aimed to investigate the occurrence of carnitine deficiency in severely malnourished patients with eating disorders during refeeding and assess its potential adverse effects on treatment outcomes.

**Method:**

In a cohort study of 56 female inpatients with eating disorders at a single hospital from March 2010 to December 2020, we measured plasma free carnitine (FC) levels and compared to those of a healthy control group (*n* = 35). The patients were categorized into three groups based on FC levels: FC deficiency (FC< 20 µmol/L), FC pre-deficiency (20 µmol/L ≤ FC< 36 µmol/L), and FC normal (36 µmol/L ≤ FC).

**Results:**

Upon admission, the patients had a median age of 26 years (interquartile range [IQR]: 21–35) and a median body mass index (BMI) of 13.8 kg/m^2^ (IQR: 12.8–14.8). Carnitine deficiency or pre-deficiency was identified in 57% of the patients. Hypocarnitinemia was associated with a decline in hemoglobin levels during refeeding (odds ratio [OR]: 0.445; 95% confidence interval [CI]: 0.214–0.926, *p* = 0.03), BMI at admission (OR: 0.478; 95% CI: 0.217–0.874, *p* = 0.014), and moderate or greater hepatic impairment at admission (OR: 6.385; 95% CI: 1.170–40.833, *p* = 0.032).

**Conclusions:**

Hypocarnitinemia, particularly in cases of severe undernutrition (BMI< 13 kg/m^2^ at admission) was observed in severely malnourished patients with eating disorders during refeeding, a critical metabolic transition phase. Moderate or severe hepatic impairment at admission was considered a potential indicator of hypocarnitinemia. Although hypocarnitinemia was not associated with any apparent adverse events other than anemia during refeeding, the possibility that carnitine deficiency may be a risk factor for more serious complications during sudden increases in energy requirements associated with changes in physical status cannot be denied. Further research on the clinical significance of hypocarnitinemia in severely malnourished patients with eating disorders is warranted.

## Abstract (plain language summary)

Carnitine is an amino acid derivative that plays an important role in the promotion and regulation of fatty acid metabolism, and carnitine deficiency is assumed in patients with anorexia nervosa associated with chronic starvation, but there are few reports on this issue. This study represents the inaugural documentation of hypocarnitinemia in severely malnourished patients with eating disorders, including anorexia nervosa. Hypocarnitinemia, particularly in cases of severe undernutrition (BMI < 13 kg/m^2^) was observed during refeeding, a critical metabolic transition phase. Moderate or severe hepatic impairment was considered a potential indicator of hypocarnitinemia. Although no apparent association with adverse events other than anemia during refeeding was identified, clinical manifestations of hypocarnitinemia may occur when a sudden increase in energy demand is added to a change in the physical condition of the patient group. Further investigation is required to determine the clinical significance of hypocarnitinemia.

## Background

Eating disorders are serious psychiatric disorders characterized by abnormal eating or weight-control behaviors that predominantly affect young women. Cognitive distortions related to weight, body shape, and eating play an important role in the development and maintenance of this disorder [1]. Among the various eating disorders, anorexia nervosa (AN) is often associated with severe physical risks due to medical comorbidities intrinsic to starvation and malnutrition, which are also associated with high mortality rates for this disease [2].

It has been reported that about 50% of patients are chronically affected with AN even after treatment [3], and this chronic starvation leads to medical complications affecting organ systems throughout the body. Starvation induces protein and fat catabolism, leading to adverse effects and atrophy of the heart, brain, liver, intestines, kidneys, and muscles [4].

Secondary carnitine deficiency is assumed in patients with AN in association with chronic starvation. Carnitine is an amino acid derivative that plays an important role in the promotion and regulation of fatty acid metabolism. In the average adult diet, approximately 75% of the daily carnitine requirement is supplied by meat and dairy products, and the remaining 25% is synthesized endogenously in the liver, kidney, and brain from lysine and methionine [5, 6]. Furthermore, 98% of the carnitine in the human body is contained in muscle [6]. Fatty acid oxidation is an important source of energy production in mammals, and during fasting periods, fatty acids are the primary substrates for energy production in liver, heart muscle, and skeletal muscle. The brain does not directly utilize fatty acids for oxidative metabolism, but rather uses ketone bodies produced by β-oxidation of fatty acids in the liver [7]. The main function of carnitine is to act as a carrier in the process of transporting long-chain fatty acids into mitochondria and excreting them out of the body as acylcarnitines (ACs) to prevent the accumulation of excess acyl compounds, which are cytotoxic in the mitochondria. Carnitine also regulates the acyl coenzyme A (CoA)/free CoA ratio in mitochondria and maintains the free CoA pool. This free CoA is used for energy production in the tricarboxylic acid circuit [8].

Carnitine deficiency can be classified into two main categories based on its underlying causes: “primary carnitine deficiency,” which is the result of genetic mutations in carnitine transporters, and “secondary carnitine deficiency,” which occurs as a consequence of underlying disease. Secondary carnitine deficiency is characterized by decreased plasma and tissue carnitine levels. It can be further categorized by its etiology, which includes: 1) congenital metabolic disorders, 2) acquired medical conditions (e.g., cirrhosis, renal failure, pregnancy and malnutrition, long-term total parenteral nutrition), or 3) medical causes (e.g., long-term hemodialysis, use of valproic acid or pivoxil-containing antimicrobial agents) [9–11].

Carnitine deficiency causes nonketotic hypoglycemia, impaired consciousness, seizures, muscle weakness, cardiomyopathy, and liver damage as a result of impaired energy production during starvation [6]. Some of these conditions are also common in patients with AN who are undernourished due to an inadequate diet, especially those with reduced muscle mass.

We searched risk factors for elevated liver enzyme (alanine aminotransferase) levels that occur with renutrition therapy during the course of treatment for severely malnourished patients with eating disorders, and found that early age of onset and a delay in the start of weight gain were associated with liver damage during refeeding [12]. Fat accumulation in hepatocytes (hepatic steatosis) due to excess glucose calories has been postulated as one of the etiologies of liver damage during refeeding, but the mechanism remains unclear. Other possible etiologies include the inhibition of fatty acid oxidation, which leads to the development of fatty liver due to carnitine deficiency associated with total parenteral nutrition [13].

With regard to AN and other eating disorders involving low body weight, the relationships among carnitine deficiency and liver damage [14, 15], myopathy [15, 16], and dilated cardiomyopathy [17] have been discussed in the literature. As for liver damage, some reports suggest an association with carnitine deficiency [14], while others do not [15]; however, no definitive conclusion has been reached. In addition, the number of reports is limited, and all have been in the form of case reports.

In the present study, we hypothesized that carnitine deficiency occurs in severely malnourished patients with eating disorders during refeeding and has adverse effects during the course of treatment, such as liver damage, hypoproteinemia, and poor weight gain. Given this background, the purpose of this study was to determine what events and symptomatology are associated with carnitine deficiency during the course of treatment.

## Methods

### Study population

The study participants were 56 patients with eating disorders who had been admitted to the Psychiatry Department of Nagoya University Hospital in Nagoya, Japan, between March 31, 2010 and December 31, 2020 for nutritional rehabilitation due to low body weight (patient group), and 35 healthy controls (control group). At the beginning of the study, 36 patients in the patient group and 20 healthy controls had already consented to participate and had specimens collected. The inclusion criteria were: 1) a diagnosis of AN-restricting type (AN-R), AN binge-eating/purging type (AN-BP), avoidant/restrictive food intake disorder, or another specified feeding or eating disorder based on the Diagnostic and Statistical Manual of Mental Disorders, Fifth Edition (DSM-5) criteria [18], 2) age ≥ 17 years, 3) recovery to at least 55% of standard body weight and no need for intensive medical management for high frequency medical complications, 4) blood and biochemical data available from two different occasions (at admission and at the time of plasma carnitine level measurement), and 5) available clinical data obtained from electronic medical records. The exclusion criteria were: 1) those who did not meet the above inclusion criteria, 2) those with obvious renal disease or undergoing dialysis, and 3) males. Two experienced psychiatrists (ST and MI) used the DSM-5 criteria to re-diagnose patients who had been diagnosed before 2013 using the previous version of the DSM (4th Edition, Text Revision).

### Refeeding protocol

Renutrition therapy at our hospital is generally performed under a structured behavioral therapy protocol with oral intake under supervision. If oral intake is insufficient, high-calorie liquid dietary supplements are used to compensate for increased caloric needs. The target body weight is discussed with the patient at the time of admission and clearly set, and the patient generally aims to reach 70–75% of standard weight, which allows for outpatient treatment. If weight gain is insufficient or hypoglycemic symptoms are unavoidable with intermittent nutrition, enteral refeeding via a nasogastric tube is additionally used. Nutritional rehabilitation is followed by a progressive increase of 200 kcal every 3 days, according to the patient’s clinical status, with an objective of 1 kg of weight gain per week. Fundamentally, renutrition therapy is based on the American Psychiatric Association guidelines [19]. The ratio of nutrients in a normal diet (1700–2300 kcal) is 15–17% protein, 23–25% lipids, and 58–62% carbohydrates.

### Study design

Plasma carnitine concentrations in both the patient and healthy groups were measured at any time after consent to participate in the study had been obtained. Blood samples were collected only once: early in the morning after fasting for the patient group during hospitalization, and at approximately 14:00 for the healthy group. We used frozen plasma samples stored at − 80 °C after centrifugation. Plasma free carnitine (FC) and total carnitine (TC) concentrations were measured using the enzyme cycling method (blood carnitine two-fraction test) [20] with Kainos reagents (Kainos Laboratories, Inc., Tokyo, Japan) and an automated analyzer (JCA-BM9130; JEOL Ltd., Tokyo, Japan). Acylcarnitine (AC) concentrations were obtained from the difference between TC and FC concentrations. The diagnosis of carnitine deficiency was based on the Japanese guideline “Diagnosis and Treatment of Carnitine Deficiency Using Diagnostic Reagents for the Blood Carnitine Bifractionation Test”, and the patient groups were then divided into three groups: A (FC < 20 µmol/L; carnitine deficiency), B (20 µmol/L ≤ FC < 36 µmol/L; very likely to develop carnitine deficiency), and C (36 µmol/L ≤ FC ≤ 74 µmol/L; normal). Classification of these three groups was based on the reference values of the Blood Carnitine Bifractionation test using the guidelines published in 2018 by the Japan Pediatric Society [21].

Basic patient information, including age at admission, age of illness onset, duration of illness, blood and biochemical data during hospitalization, and progress of nutritional therapy, were obtained retrospectively by electronic chart review. Blood and biochemical data were obtained twice: (1) at the time of admission (before the start of renutrition therapy), and (2) at the time of plasma carnitine level measurement (after the start of renutrition therapy), when the patients were above 55% of standard body weight and out of the acute phase during which medical physical management was given top priority. The clinical parameters analyzed included the duration of hospitalization, BMI at admission, BMI at discharge, weight gain ratio (BMI at discharge / BMI at admission), nadir BMI during hospitalization, time to nadir BMI, time to carnitine measurement, initial prescribed calories, prescribed calories at plasma carnitine concentration measurement, maximum prescribed calories, total protein (TP), albumin, glucose, blood urea nitrogen, creatinine, aspartate aminotransferase (AST), alanine aminotransferase (ALT), lactate dehydrogenase, alkaline phosphatase, gamma glutamyl transferase (GGT), white blood cell count, red blood cell (RBC) count, hemoglobin, hematocrit, platelet, triglyceride, uric acid, sodium, potassium, chloride, and phosphate levels. This study was approved by the Ethics Review Committee of the Nagoya University Graduate School of Medicine (2009 − 0853) and conducted in accordance with all relevant guidelines.

### Statistical analysis

Univariate analysis was used to describe the sample, including frequency (%), range, and median (interquartile range [IQR]). We used the Mann–Whitney *U* test and the Steel–Dwass test to compare exploratively the median of continuous variables (such as age), and Fisher’s exact test to compare exploratively the proportions of categorical variables (such as disease type) between groups. The effect size was reported in *r*. Multivariate logistic regression analysis was performed with the presence of carnitine deficiency as the dependent variable. The independent variables included BMI (at admission or at the time of carnitine level measurement), the presence of hypoproteinemia and liver damage (which were considered clinically important), and factors identified in the univariate analysis. All *p* values < 0.05 were considered statistically significant. All analyses were performed using JMP Pro 16 software (SAS Institute, Cary, NC, USA).

## Results

### Comparison of the patients and healthy controls

**Fig. 1:**
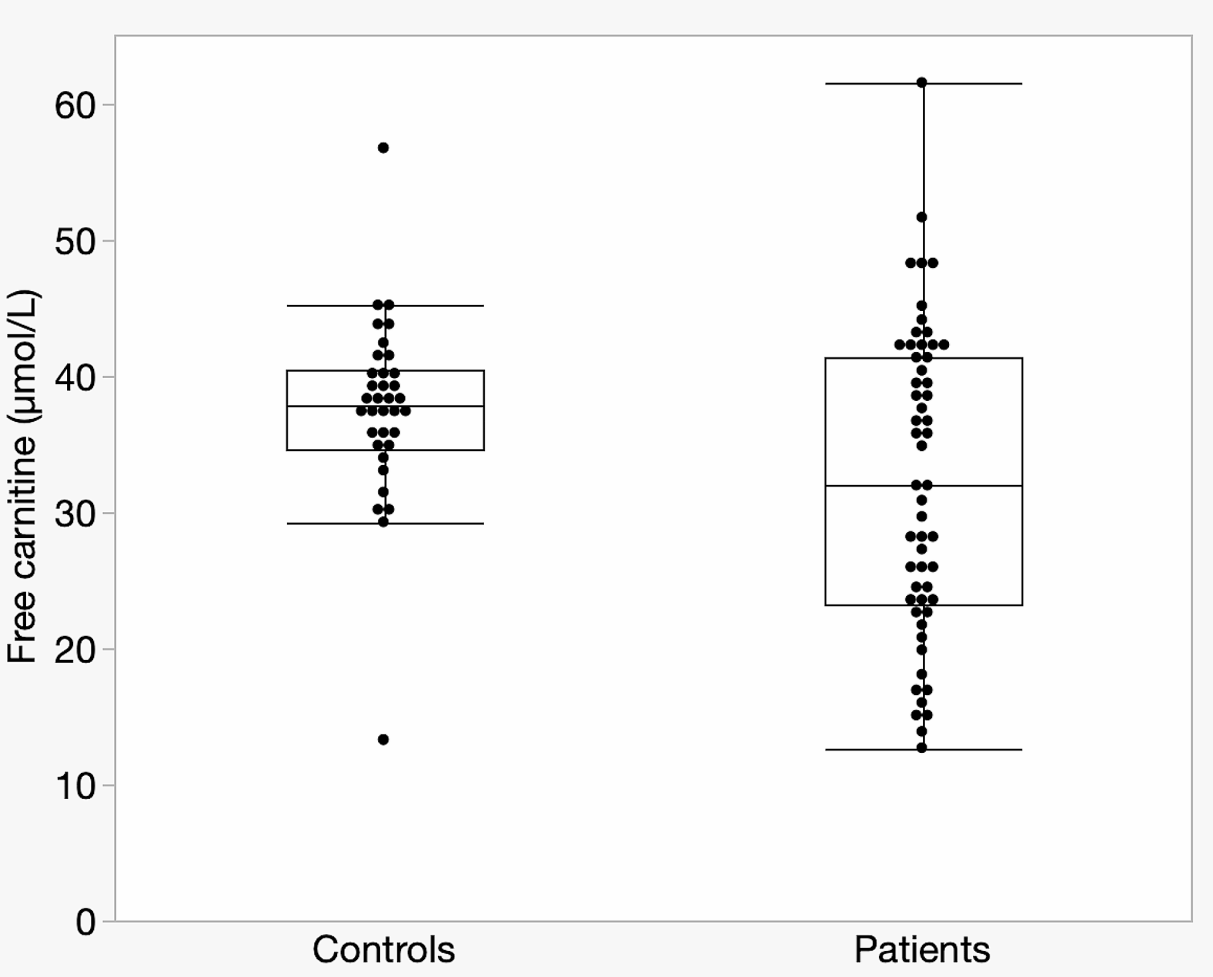
Concentrations of free carnitine in plasma by group

Fifty-six patients and 35 healthy controls participated in the study. The distribution of FC levels in the two groups is shown below (Fig. 1). The median FC levels were significantly lower in the patient group than in the healthy controls (32.0 µmol/L vs. 37.8 µmol/L, respectively; *p* = 0.0249, *r* = 0.24). The percentage of patients with FC concentrations below the lower reference limit (36 µmol/L) was 57% in the patient group, compared with 34% in the healthy controls. In addition to FC, BMI, AC, TC, and AC/FC were all significantly lower in the patients than in the healthy controls (Table 1).

**Table 1:** Comparison between the patient groups and controls

	Controls (*N* = 35)		Patients (*N* = 56)			
	Median (IQR)	Range	Median (IQR)	Range	*p* value^a^	*r*
Age (years)	24 (21–39)	20–49	26 (21–35)	17–53	0.7655	0.03
Height (m)	1.58 (1.546–1.611)	1.43–1.73	1.559 (1.528–1.597)	1.425–1.67	0.0976	0.17
Weight (kg)	49.5 (46.6–59)	42.2–85	30.1 (26.6–34.8)	18.9–44.5	< 0.0001	0.83
BMI at time of carnitine measurement (kg/m^2^)	20 (18.8–22.6)	16.6–34.2	13.8 (12.8–14.8)	10.8–18	< 0.0001	0.83
Free carnitine (36–74 µmol/L)	37.8 (34.7–40.5)	13.3–56.8	32.0 (23.2–41.5)	12.7–61.6	0.0249	0.24
Acylcarnitine (6–23 µmol/L)	7 (5.7–9.1)	3.0–11.3	3.8 (2.5–5.8)	0.7–9.9	< 0.0001	0.59
Total carnitine (45–91 µmol/L)	44.7 (41–48.9)	20.6–67.6	36.3 (26.1–46.2)	13.4–71.1	0.0012	0.34
Acylcarnitine/free carnitine (AC/FC)	0.19 (0.16–0.24)	0.07–0.55	0.12 (0.1–0.16)	0.04–0.27	< 0.0001	0.57

IQR, interquartile range; BMI, body mass index; ^a^Mann–Whitney *U* test

### Patients’ characteristics

The characteristics of the patient group are shown in Table 2. These patients had a median age of 26 years (IQR: 21–35), a median BMI at admission of 12.4 kg/m^2^ (IQR: 11.1–14.2), a median minimum BMI during hospitalization of 12 kg/m^2^ (IQR: 10.7–13.7), and a median BMI at discharge of 14.8 kg/m^2^ (IQR: 14–15.8). The median number of days to the date of plasma carnitine level measurement was 28, which is to say that the studies were conducted at around 1 month after admission. By disease type, AN-R and AN-BP accounted for about 40%, respectively.

**Table 2:** Patients’ characteristics and blood biochemical findings at the time of admission (*N* = 56)

	Median (IQR)	Range
Age (years)	26 (21–35)	17–53
Age of illness onset (years)	20 (15.3–21.8)	11–39
Duration of illness (years)	6.7 (2–11.8)	0.6–28
Duration of hospitalization (days)	50 (35–77)	19–123
BMI at admission (kg/m^2^)	12.4 (11.1–14.2)	8.9–17.2
BMI at discharge (kg/m^2^)	14.8 (14–15.8)	12.5–19.0
Ratio of BMI increase^a^	1.17 (1.1–1.3)	0.95–1.58
Minimum BMI during hospitalization (kg/m^2^)	12 (10.7–13.7)	8.9–17.0
Time to nadir BMI (days)	0 (0–7)	0–16
Time to measure carnitine level (days)	28 (16–41)	7–74
Initial prescribed calories (kcal/day)	980 (750–1480)	480–1700
Prescribed calories when measuring carnitine levels (kcal/day)	2,010 (1,700–2,370)	1,000–3,125
Maximum prescribed calories (kcal/day)	2,300 (2,020–2,600)	1,600–3,625
TP (6.7–8.3 g/dL)^b^	6.6 (6.1–7)	4.6–8.2
Albumin (4.1–5 g/dL)	4.1 (3.5–4.7)	2.1–5.5
Glucose (73–109 mg/dL)	77.5 (71–84)	48–132
BUN (8–22 mg/dL)	13.6 (10.1–18.6)	4.1–41.7
Creatinine (0.4–0.7 mg/dL)	0.63 (0.52–0.75)	0.29–3.04
AST (13–33 IU/L)	32.5 (23–54.8)	13–2881
ALT (6–27 IU/L)	27.5 (18–69)	9–2751
LDH (119–229 IU/L)	204 (172.2–284)	112–746
ALP (115–359 IU/L)	191 (135.8–298.5)	83–757
GGT (10–47 IU/L)	29 (19.3–73.3)	11–748
WBC (3.8–8.5×10^3^/µL)	3.75 (2.9–5.18)	1.4–9.6
RBC (3.6–5.0×10^6^/µL)	3.97 (3.35–4.38)	2.02–5.47
Hemoglobin (11–16 g/dL)	12.2 (10.5–13.7)	7.4–16.4
Hematocrit (32–48%)	36.1 (31.8–40)	22.3–47.3
Platelets (160–410×10^3^/µL)	207.5 (150.5–285.5)	49–472
Triglyceride (30–149 mg/dL)	78 (61.3–116.3)	17–310
Uric acid (2.3–7 mg/dL)	4 (3.5–5.4)	0.9–12.2
Sodium (138–146 mEq/L)	140 (138–142)	124–145
Potassium (3.6–4.9 mEq/L)	3.8 (3.3–4.3)	2.4–5.6
Chloride (99–109 mEq/L)	102.5 (99–108)	68–110
Phosphate (2.5–4.7 mEq/L)	3.65 (3.2–3.9)	0.6–5.1

	Frequency (%)	
AN-R	21 (38)	
AN-BP	22 (39)	
ARFID	7 (13)	
OSFED	6 (10)	
Low TP (< 6.7 g/dL)	30 (54)	
Low albumin (< 4.1 g/dL)	25 (45)	
Low glucose (< 73 mg/dL)	18 (32)	
Elevated ALT (> 27 U/L)	30 (54)	

IQR, interquartile range; BMI, body mass index; TP, total protein; BUN, blood urea nitrogen; AST, aspartate aminotransferase; ALT, alanine aminotransferase; LDH, lactate dehydrogenase; ALP, alkaline phosphatase; GGT, gamma glutamyl transferase; WBC, white blood cells; RBC, red blood cells; AN-R, anorexia nervosa-restricting type; AN-BP, anorexia nervosa-binge-eating/purging type; ARFID, avoidant/restrictive food intake disorder; OSFED, other specified feeding or eating disorder; ^a^Rate of BMI increase, BMI at discharge / BMI at admission; ^b^Reference range shown in parentheses

### Hematological and biochemical findings at admission in the patient group

The hematological and biochemical findings of the patient group at admission are shown in Table 2. Hypoproteinemia and hypoalbuminemia were present in about half of the patients; 54% of the patients had hepatic involvement, of which, 13 of 56 (23%) had moderate or severe hepatic involvement (described later, Table 3).

**Table 3:** Comparison of Groups A, B, and C by carnitine concentration (characteristics and blood biochemical findings at the time of admission)

	Group A (*N* = 9)			Group B (*N* = 23)		Group C (*N* = 24)				
	FC < 20 (µmol/L)			20 ≤ FC < 36 (µmol/L)		36 ≤ FC (µmol/L)				
	Median (IQR)		Range	Median (IQR)	Range	Median (IQR)	Range	*p* value^a^		*r*
Age (years)	31 (22–46)		21–51	27 (22–34)	18–48	25 (20–36)	17–53	n.s.^b^		
Age of illness onset (years)	19 (16–24)		15–30	20 (15–21)	11–39	20 (16–22)	14–38	n.s.		
Duration of illness (years)	11 (4–23.7)		0.7–26	9 (2–11)	0.6–22	5.5 (1.3–11)	0.7–28	n.s.		
Duration of hospitalization (days)	79 (60–107)		30–122	50 (37–70)	26–104	46 (28–71)	19–123	0.0473^c^		0.32
								0.0444^d^		0.32
BMI at admission (kg/m^2^)	10.8 (10–12.1)		8.9–12.1	12 (11.5–13.5)	9.3–16.7	13.3 (11.8–14.6)	9.7–17.2	0.0049^c^		0.42
								0.0398^d^		0.33
BMI at discharge (kg/m^2^)	14.3 (13.8–15.1)		13.3–15.4	14.7 (13.8–15.6)	12.8–17.2	15.3 (14.6–16.2)	12.8–19	n.s.		
Ratio of BMI increase^e^	1.37 (1.2–1.46)		0.95–1.58	1.13 (1.09–1.25)	0.95–1.58	1.17 (1.06–1.31)	0.95–1.51	0.0467^d^		0.32
Minimum BMI during hospitalization (kg/m^2^)	10.5 (9.4–11.3)		8.9–12	12.4 (11–13.5)	9.1–15.7	13.2(11.7–14.4)	9.7–17	0.0026^c^		0.44
								0.0147^d^		0.37
Time to nadir BMI (days)	3 (0–6.5)		0–10	2 (0–7)	0–16	4 (0.3–7)	0–13	n.s.		
Initial prescribed calories (kcal/day)	500 (500–1,450)		480–1,700	960 (750–1,200)	500–1,700	1,175 (850–1,520)	750–1,700	n.s.		
Prescribed calories when measuring carnitine levels (kcal/day)	2,125 (1,960–2,500)		1,520–2,600	2,000 (1,600–2,300)	1,440–2,700	2,090 (1,800–2,394)	1,000–3,125	n.s.		
Maximum prescribed calories (kcal/day)	2,400 (2,275–2,525)		1,920–2,600	2,300 (2,000–2,600)	1,600–3,625	2,300 (2,123–2,988)	1,600–3,425	n.s.		
TP (6.7–8.3 g/dL)	5.5 (4.8–6.3)		4.8–6.3	6.6 (5.8–7)	4.8–7.9	6.8 (6.3–7.3)	5.6–8.2		0.0032^c^	0.44
									0.0266^d^	0.35
Albumin (4.1–5 g/dL)	3.3 (2.8–3.8)		2.7–3.9	4.1 (3.6–4.8)	2.2–5	4.5 (4–4.9)	2.1–5.5		0.0045^c^	0.42
									0.0058^d^	0.41
Glucose (73–109 mg/dL)	72 (61–80)		51–85	77 (70–83)	48–102	81 (74–87)	57–132		n.s.	
BUN (8–22 mg/dL)	15 (13–17.9)		10.9–24	12.3 (9.4–22)	4.1–36.9	13.3 (9.6–16)	6–41.7		n.s.	
Creatinine (0.4–0.7 mg/dL)	0.65 (0.35–0.76)		0.29–0.89	0.66 (0.57–0.81)	0.37–1.2	0.62 (0.56–0.71)	0.37–3.04		n.s.	
AST (13–33 IU/L)	96 (45–150)		29–228	30 (20–39)	15–2881	31 (20–40)	13–137		0.0068^c^	0.41
									0.0129^d^	0.38
ALT (6–27 IU/L)	96 (33–342)		22–455	29 (18–44)	9–2751	24 (15–44)	10–130		0.0145^c^	0.37
LDH (119–229 IU/L)	308 (200–521)		150–746	195 (156–265)	127–622	197 (173–251)	112–370		n.s.	
ALP (115–359 IU/L)	299 (165–539)		104–757	178 (104–247)	83–490	182 (142–307)	113–506		n.s.	
GGT (10–47 IU/L)	59 (32–122)		22–263	26 (17–39)	11–318	27 (18–90)	14–748		0.0494^d^	0.31
WBC (3.8–8.5×10^3^/µL)	3.4 (2.2–5.5)		1.9–7.3	3.6 (2.9–5.2)	2.1–9.6	4 (2.9–5.3)	1.4–7.1		n.s.	
RBC (3.6–5.0×10^6^/µL)	3.6 (3–4.9)		2.6–5.2	4.1 (3.5–4.3)	2–5.5	4 (3.2–4.4)	2.5–5.1		n.s.	
Hemoglobin (11–16 g/dL)	11.9 (9.8–15.9)		8.8–16.4	12.2 (10.5–13.6)	7.4–16.4	12.4 (10.5–13.6)	8.1–16		n.s.	
Hematocrit (32–48%)	34 (29.6–45.6)		27.6–46.1	35.6 (32.7–39.4)	22.3–47.3	36.4 (31.8–40.8)	24.5–46.3		n.s.	
Platelets (160–410×10^3^/µL)	196 (94–348)		59–472	218 (159–304)	107–393	203 (160–269)	109–396		n.s.	
Triglyceride (30–149 mg/dL)	61 (43–97)		34–120	76 (61–120)	17–188	91 (73–138)	41–310		n.s.	
Uric acid (2.3–7 mg/dL)	3.9 (3–4.4)		0.9–7.1	4.2 (3.4–5.4)	1.3–10.6	4.1 (3.8–5.6)	3.8–5.6		n.s.	
Sodium (138–146 mEq/L)	139 (135–142)		135–142	139 (137–142)	124–145	140 (139–142)	132–143		n.s.	
Potassium (3.6–4.9 mEq/L)	3.7 (3.1–4.3)		2.4–5.4	3.8 (3.2–4.3)	2.8–5.6	3.9 (3.5–4.3)	2.4–5		n.s.	
Chloride (99–109 mEq/L)	100 (98–107)		94–107	103 (98–107)	88–110	103 (100–105)	68–108		n.s	
Phosphate (2.5–4.7 mEq/L)	3 (2.8–3.7)		1.9–3.9	3.8 (3.2–3.9)	0.6–5.1	3.6 (3.4–3.9)	2.9–5.1		0.0467^c^	0.25

	Group A (*N* = 9)			Group B + C (*N* = 47)						
	FC < 20 (µmol/L)			20 ≤ FC (µmol/L)						
	Frequency (%)							*p* value^f^		
Poor weight gain^g^	0 (0)			31 (66)					n.a.^i^	
Delayed start of weight gain^h^	6 (67)			31 (66)					n.a.	
Low TP (< 6.7 g/dL)	8 (89)			22 (47)					0.0286	
Low albumin (< 4.1 g/dL)	9 (100)			16 (34)					< 0.0001	
Low glucose (< 73 mg/dL)	5 (56)			13 (28)					0.1288	
ALT ≥ 81 (IU/L)^j^	6 (67)			7 (15)					0.003	
Low phosphate (< 2.5 mEq/L)	1 (11)			2 (4)					0.415	

FC, free carnitine; IQR, interquartile range; BMI, body mass index; TP, total protein; BUN, blood urea nitrogen; AST, aspartate aminotransferase; ALT, alanine aminotransferase; LDH, lactate dehydrogenase; ALP, alkaline phosphatase; GGT, gamma glutamyl transferase; WBC, white blood cells; RBC, red blood cells; ^a^Steel–Dwass test; ^b^n.s., not significant; ^c^difference between groups A and C; ^d^significant difference between groups A and B; ^e^Rate of BMI increase, BMI at discharge / BMI at admission; ^f^Fisher’s exact test; ^g^Rate of BMI increase (BMI at discharge / BMI at admission) < 1; ^h^Time to nadir BMI ≥ 1 (days); ^i^n.a. not assessable; ^j^More than three times the reference value

### Comparison of the A, B, and C groups

Patients were divided into three groups according to plasma FC concentration: A (*n* = 9; FC < 20 µmol/L; carnitine deficiency group), B (*n* = 23; 20 µmol/L ≤ FC < 36 µmol/L; carnitine pre-deficiency group), and C (*n* = 24; 36 µmol/L ≤ FC; normal group) (Tables 3 and 4). Of the 56 patients, 32 (57%) showed decreased carnitine levels, including those with carnitine and pre-carnitine deficiency. As for the patients’ characteristics (Table 3), BMI at admission and minimum BMI were significantly lower in group A than in group C. The duration of hospitalization and weight gain ratio were significantly higher in group A than in group C. This may be related to the fact that the patients in group A were underweight at the time of admission compared with the other groups. No significant differences in age, age of illness onset, duration of illness, time to nadir BMI, or amount of prescribed calories were observed among the three groups.

**Table 4:** Comparison of Groups A, B, and C by carnitine concentration (clinical and blood biochemical findings at the time of carnitine level measurement)

	Group A (*N* = 9) FC < 20 (µmol/L)		Group B (*N* = 23) 20 ≤ FC < 36 (µmol/L)		Group C (*N* = 24) 36 ≤ FC (µmol/L)			
	Median (IQR)	Range	Median (IQR)	Range	Median (IQR)	Range	*p* value^a^	*r*
Time to measure carnitine level (days)	49 (27–65)	16–74	28 (18–34)	7–66	21 (14–31)	10–71	0.0424^b^	0.32
BMI at time of carnitine measurement (kg/m^2^)	13.1 (12.6–13.9)	12.4–14.8	13.8 (12.6–14.8)	10.8–17.2	14.4 (13.1–15.3)	11.3–18	n.s.	
TP (6.7–8.3 g/dL)	6 (5.4–6.3)	4.3–7.4	6.2 (5.6–6.5)	5.1–6.9	6.6 (6.2–7)	5.5–7.7	0.0258^b^	0.35
							0.0177^e^	0.36
Albumin (4.1–5 g/dL)	3.4 (2.8–3.7)	2.3–4.3	3.8 (3.4–4.2)	2.9–4.5	4.1 (3.8–4.5)	2.1–5	0.0104^b^	0.39
Glucose (73–109 mg/dL)	76 (69–79)	65–82	77 (73–80)	60–85	80 (74–95)	64–132	n.s.^d^	
BUN (8–22 mg/dL)	14.4 (10.9–18.5)	8–21.9	11 (9–18)	4–41.1	12.6 (10.4–15.9)	7.3–33.6	n.s.	
Creatinine (0.4–0.7 mg/dL)	0.46 (0.3–0.62)	0.22–0.86	0.56 (0.48–0.64)	0.39–0.79	0.57 (0.5–0.7)	0.41–1.12	n.s.	
AST (13–33 IU/L)	24 (19–35)	16–133	30 (19–38)	14–61	26 (21–34)	17–50	n.s.	
ALT (6–27 IU/L)	27 (15–47)	12–170	30 (20–68)	9–2751	28 (20–44)	12–91	n.s.	
LDH (119–229 IU/L)	243 (203–306)	146–309	177 (158–237)	127–622	193 (160–235)	123–310	n.s.	
ALP (115–359 IU/L)	295 (133–384)	107–597	165 (125–247)	94–452	182 (144–276)	94–387	n.s.	
GGT (10–47 IU/L)	29 (20–38)	13–75	25 (19–58)	13–86	29 (17–57)	13–216	n.s.	
WBC (3.8–8.5×10^3^/µL)	4.2 (3.4–5)	1.9–5.6	3.6 (2.8–4.3)	1.8–6.9	4.2 (3.7–5.3)	1.4–7.1	n.s.	
RBC (3.6–5.0×10^6^/µL)	3.21 (2.84–3.52)	2.4–3.76	3.67 (3.2–4.1)	2.5–4.8	3.86 (3.5–4.1)	2.55–4.64	0.0101^b^	0.39
Hemoglobin (11–16 g/dL)	10.3 (8.6–10.7)	8.1–11.1	11.1 (10–12.2)	8.5–13.2	12.1 (10.9–12.9)	8.5–14.5	0.0021^b^	0.45
Hematocrit (32–48%)	31.4 (27.9–33.3)	23–35	34.3 (30.6–37.7)	27.1–39.4	36.5 (32.7–39.2)	26.4–41.8	0.0078^b^	0.4
Platelets (160–410×10^3^/µL)	310 (99–399)	160–566	222 (146–254)	106–301	222 (177–272)	145–516	0.0357^c^	0.33
Triglyceride (30–149 mg/dL)	80 (48–101)	35–135	69 (48–82)	38–153	64 (49–120)	36–310	n.s.	
Uric acid (2.3–7 mg/dL)	4.1 (3.6–5.2)	2.8–5.5	3.3 (2.8–4.2)	1.5–6.7	4.5 (3.8–5.4)	3.2–11.4	0.0021^e^	0.45
Sodium (138–146 mEq/L)	142 (140–143)	135–144	142 (141–144)	128–145	141 (140–142)	135–143	n.s.	
Potassium (3.6–4.9 mEq/L)	4.3 (4.1–4.4)	3.8–4.7	4.3 (4–4.5)	3–4.9	4.2 (4–4.6)	3.5–5.2	n.s.	
Chloride (99–109 mEq/L)	106 (104–108)	100–109	106 (105–107)	95–116	101 (103–107)	99–110	n.s.	
Phosphate (2.5–4.7 mEq/L)	4.6 (3.9–5.2)	3.6–5.3	4.1 (3.6–4.6)	2.5–5.6	4.4 (4–4.6)	3.5–5.6	n.s.	

	Group A (*N* = 9)		Group B + C (*N* = 47)					
	FC < 20 (µmol/L)		20 ≤ FC (µmol/L)					
	Frequency (%)						*p* value^f^	
Low albumin (< 4.1 g/dL)	8 (89)		22 (47)				0.0307	
Elevated ALT (> 27 U/L)	4 (44)		27 (57)				0.4928	
Low hemoglobin (< 11 g/dL)	9 (100)		24 (51)				0.0073	

FC, free carnitine; IQR, interquartile range; BMI, body mass index; TP, total protein; BUN, blood urea nitrogen; AST, aspartate aminotransferase; ALT, alanine aminotransferase; LDH, lactate dehydrogenase; ALP, alkaline phosphatase; GGT, gamma glutamyl transferase; WBC, white blood cells; RBC, red blood cells; ^a^Steel–Dwass test; ^b^significant difference between groups A and C; ^c^significant difference between groups A and B; ^d^n.s. not significant; ^e^significant difference between groups B and C; ^f^Fisher’s exact test

Blood biochemical findings at admission are shown in Table 3. TP, albumin, and phosphate were significantly lower in group A than in group C, suggesting that the patients in group A were more undernourished than those in the other groups. AST, ALT, and GGT were significantly elevated in group A compared with group C, indicating that liver injury was more prominent in group A. Blood biochemical findings at the time of carnitine measurement are shown in Table 4. TP, albumin, RBC, hemoglobin, and hematocrit were significantly lower in group A than in group C, and the patients in group A remained more undernourished after the start of renutrition therapy than did those in the other groups; the patients in group A also developed anemia that was not present at admission. The decrease in BMI and increase in liver enzymes seen in group A at admission improved.

### Findings related to hypocarnitinemia

Findings related to hypocarnitinemia (FC < 20 µmol/L) during hospitalization are shown in Tables 3 and 4. Hypoproteinemia and hypoalbuminemia at admission were significantly associated with hypocarnitinemia (Table 3). We defined “moderately or greater elevated ALT levels” as an increase in ALT levels of more than three times the normal value (ALT ≥ 81 IU/L) in accordance with a previous report [22]. Moderately or greater elevated ALT levels at admission were significantly associated with hypocarnitinemia. No association between hypocarnitinemia and poor weight gain or delay in the start of weight gain was observed (Table 3). Furthermore, both hypoalbuminemia and decreased hemoglobin levels at the time of carnitine measurement were significantly associated with hypocarnitinemia (Table 4).

### Events involving carnitine deficiency

To identify the most important risk factors for hypocarnitinemia (FC < 20 µmol/L) during refeeding, multivariate logistic regression analysis was performed using the following factors, which were significantly different in the univariate analysis after dividing the patients into three groups according to plasma FC levels (Table 5): (1) two variables at admission: BMI and the presence of elevated ALT (≥ 81 IU/L), and (2) three variables at the time of carnitine level measurement: BMI, the presence of low albumin, and hemoglobin level. Among the clinical parameters, the presence of low albumin was selected as an indicator of nutritional status, and hemoglobin level, which was clinically easy to interpret, was selected as an indicator of anemia. The results showed that BMI at admission (odds ratio [OR]: 0.478; 95% confidence interval [CI]: 0.217–0.874; *p* = 0.014), increased ALT level at admission (≥ 81 IU/L; OR: 6.385; 95%CI: 1.170–40.833; *p* = 0.032), and hemoglobin level at carnitine measurement (OR: 0.445; 95%CI: 0.214–0.926; *p* = 0.03) were significantly associated with the risk of hypocarnitinemia during refeeding.

**Table 5:** Odds ratios (95% confidence intervals) for determinants of hypocarnitinemia (free carnitine level < 20 mol/L)

Variable (at the time of admission)		*p* value
BMI (kg/m^2^)	0.478 (0.217–0.874)	0.014
ALT (≥ 81 IU/L)^a^	6.385 (1.170–40.833)	0.032

Variable (at the time of carnitine level measurement)		
BMI (kg/m^2^)	0.942 (0.496–1.790)	0.855
Low albumin (< 4.1 g/dL)	2.201 (0.187–25.948)	0.531
Hemoglobin (g/dL)	0.445 (0.214–0.926)	0.03

BMI, body mass index; ALT, alanine aminotransferase; ^a^More than three times the reference value

## Discussion

In the present study, we aimed to investigate the presence of secondary carnitine deficiency in severely malnourished patients with eating disorders and to determine the associated events and symptomatology. The results indicated that, as hypothesized, despite undergoing renutrition therapy, the patient group had hypocarnitinemia, and this was associated with BMI at admission, moderate or greater hepatic impairment at admission, and anemia during refeeding. However, contrary to our hypothesis, no association was observed with hepatic impairment during refeeding, poor weight gain over the course of treatment, or delayed onset of weight gain.

To our knowledge, only a few case reports have focused on hypocarnitinemia in patients with AN or other eating disorders with malnutrition [14–16]. We believe that the present report is the first clinical research to identify and report hypocarnitinemia in such patients.

### Hypocarnitinemia at metabolic transition

In the present study, 57% of underweight patients with eating disorders were found to be carnitine- or pre-carnitine-deficient at approximately 1 month (median) after the initiation of renutrition therapy. In a previous study, Okamoto et al. [15] also reported a case of hypocarnitinemia during the recovery phase in a patient with AN. In the present study, the carnitine deficiency (group A) and pre-deficiency (group B) groups were severely undernourished patients with BMIs of 10.8 kg/m^2^ (median) and 12 kg/m^2^ (median), respectively, and both had a BMI < 13 kg/m^2^ at admission (Table 3). As reported by Kawai et al. [23], when BMI is < 13 kg/m^2^, protein, not fat, is the main source of energy, and protein catabolism is considered to be increased. In the case of septic patients, in addition to a relative lack of carnitine due to high hypermetabolism, protein catabolism may reduce carnitine production, resulting in carnitine deficiency [9]. Although the patients in the present study were recovering from starvation and had different diseases and pathologies, they shared the same hypermetabolic state in addition to increased protein catabolism, and it is possible that a similar mechanism may be responsible for carnitine deficiency.

During refeeding, the body undergoes a metabolic transition from catabolism to anabolism [24]. In addition, during the period of weight gain in AN, basal metabolism is suppressed and lean body mass synthesis predominates, so it is important to manage the body leading to the synthesis of lean tissue during this period [23]. In the present study, carnitine deficiency was observed in more than half of the patients with eating disorders who were underweight, even at around 1 month after the start of renutrition therapy. This may suggest that it takes more than a month for protein anabolism to stabilize, and with it, carnitine levels.

Furthermore, in AN, caloric requirements increase dramatically as renutrition therapy progresses, i.e., as the metabolism recovers from starvation [24]. The patients with eating disorders in the present study were accompanied by severe emaciation, with a BMI < 13 kg/m^2^ (median 12.4) at admission, suggesting that they had considerably reduced muscle mass and depleted carnitine stores. Considering that the measurement of carnitine levels during the renutrition treatment process was performed at a time of increased metabolism due to improved nutritional status, it is possible that an imbalance between carnitine biosynthesis, carnitine intake, and carnitine demand may have caused the carnitine decline.

### Association with anemia during refeeding

In the present investigation, an association was observed between hypocarnitinemia and anemia during refeeding. With regard to the connection between hypocarnitinemia and anemia, it has been reported that carnitine deficiency is one of the factors involved in the development of erythropoietin-resistant anemia in patients undergoing dialysis [25]. Carnitine is involved in stabilizing erythrocyte membranes, and its deficiency increases erythrocyte membrane fragility and shortens the erythrocyte life-span [26, 27]. In addition, l-carnitine supplementation has been reported to improve anemia and reduce the requirement for erythropoiesis-stimulating agents [28].

In the case of AN, it has been reported that hyperlipidemia occurs during refeeding and that l-carnitine administration improves the rapid decline in hematocrit [29]. Although we did not observe apparent refeeding syndrome (Crook, 2014) or hyperlipidemia in group A (carnitine deficiency group) in the present study, the clinical picture of abnormal fatty acid metabolism and the consequent development of anemia is similar to previous reports, suggesting the involvement of a common etiology in terms of carnitine deficiency (relative deficiency in the previous report and absolute deficiency in our cohort).

### Association with BMI at admission

In the present study, hypocarnitinemia during refeeding was associated with BMI at admission. On the other hand, no association with BMI was observed at the time carnitine levels were measured. In other words, hypocarnitinemia was associated with BMI at admission, the period of highest undernutrition.

It has been reported that undernutrition is a factor in secondary carnitine deficiency [10]. Reduced plasma carnitine levels have been reported in children with low protein-calorie nutrition, such as those with kwashiorkor and marasmus [30, 31], as has a decrease in serum carnitine levels in undernourished patients with impaired oral intake [32].

As mentioned earlier, 9 patients in group A (carnitine deficiency) and 23 patients in group B (carnitine pre-deficiency) were in a state of carnitine decline (57%), and the median BMI at admission was < 13 kg/m^2^ in both groups. In other words, the results of this study indicate that more than half of the severely malnourished patients with a BMI < 13 kg/m^2^ had carnitine deficiency or pre-deficiency. Therefore, the possibility of carnitine deficiency should be considered in cases of low BMI at admission.

### Association with liver damage at admission

The present study revealed that hypocarnitinemia during refeeding was associated with moderate or greater hepatic impairment at admission. On the other hand, no significant difference in liver function was found between the three groups at the time of hypocarnitinemia, that is, after the start of refeeding, and liver function was almost normalized. In other words, no association was found between hypocarnitinemia and liver damage. It is thought that moderate or severe hepatic impairment at admission predicts hypocarnitinemia during refeeding in the subsequent course of the disease.

The liver is one of the organs capable of biosynthesizing carnitine. The association between hypocarnitinemia and liver damage in AN has been reported by Fukusako et al. [14], and assumes a carnitine deficiency secondary to impaired carnitine production due to liver damage associated with undernutrition. Similarly, based on our results, we hypothesize that liver biosynthesis of carnitine may have been decreased due to liver injury at the time of admission, resulting in hypocarnitinemia during refeeding.

Patients with AN often show liver damage associated with undernutrition, and a multifactorial etiology has been hypothesized: nutritional hepatitis with fatty changes [33], ischemic hepatitis associated with dehydration and circulatory failure [34, 35], and starvation-induced autophagy [36, 37]. Low body weight is a risk factor for all of these etiologies [38–42]. Although liver damage is often mild to moderate and gradually resolves with weight gain, the course of such an injury should be carefully monitored because, as shown in the present study, moderate or severe liver damage at admission may predict hypocarnitinemia during refeeding.

### Clinical significance of hypocarnitinemia in severely malnourished patients with eating disorders

The present study revealed a large percentage of potential secondary carnitine deficiency during refeeding in severely malnourished patients with eating disorders. Carnitine deficiency was associated with anemia, but not with liver damage, poor weight gain, or other apparent adverse events. The bioavailability of carnitine is also said to be increased in vegetarians [5, 43]. Therefore, in vegetarians, it is possible that low carnitine levels are compensated for and homeostasis is maintained to some extent. On the other hand, there have been reports of an association between secondary carnitine deficiency and cardiomyopathy [44]. It is possible that when a highly undernourished patient with AN suddenly reduces his or her caloric intake or has a sudden increase in energy demand due to complications such as infection or diarrhea, carnitine homeostasis may be disrupted and clinical signs of masked hypocarnitinemia may develop, and this may be associated with the high mortality rate of patients with AN.

### Limitations

This study has some limitations. First, as this was a retrospective study, causality could not be proven. Second, the sample size was small, the study was limited to women only, and to severe or hospitalized cases, so the results cannot be generalized to outpatient or community samples. Moreover, we cannot rule out the effect of carnitine deficiency in nutritional supplements. During refeeding, especially during the initial period of significant low body weight, 36 (64%) of 56 patients were found to have used non-carnitine-containing nutritional supplements (alone or in combination with meals). Therefore, the extent to which insufficient carnitine intake affected the development of hypocarnitinemia in individual cases remains unclear. Moreover, several drugs may contribute to increased renal carnitine excretion and hypocarnitinemia. Among the 56 patients, three were taking sodium valproate (400 mg/day). No statistical association with hypocarnitinemia was found, but it is conceivable that this could have affected the laboratory values. There was no concomitant use of pivoxil-containing antimicrobials or other potentially causative agents.

## Conclusions

In the present study, hypocarnitinemia was observed during refeeding, i.e., during a metabolic transition, among malnourished patients with eating disorders. This hypocarnitinemia was associated with anemia during refeeding. Our findings suggest that patients severely undernourished with a BMI < 13 kg/m^2^ at admission and those with moderate or severe hepatic impairment at admission may be at risk of hypocarnitinemia. On the other hand, hypocarnitinemia was not associated with any apparent adverse events other than anemia. However, there have been reports of an association between hypocarnitinemia and cardiomyopathy, and the possibility that carnitine deficiency may be a risk factor for more serious complications during sudden increases in energy requirements associated with changes in physical status cannot be denied. Thus, further investigation of the possible role of hypocarnitinemia in the pathogenesis of more severe cases and those with sudden changes in physical and medical status is warranted.

It is hoped that the results of this exploratory study will help to promote further research on the clinical significance of hypocarnitinemia in patients with eating disorders and the need for carnitine replacement therapy.

## Data Availability

The data that support the findings of the study are available from the corresponding author upon reasonable request. The data are not publicly available due to privacy or ethical restrictions.

## Abbreviations

FC: Free carnitine
AC: Acylcarnitine
IQR: Interquartile range
BMI: Body mass index
OR: Odds ratio
CI: Confidence interval
AN: Anorexia nervosa
AN-R: Anorexia nervosa-restricting type
AN-BP: Anorexia nervosa-binge-eating/purging type
ARFID: Avoidant/restrictive food intake disorder
OSFED: Other specified feeding or eating disorder
DSM-5: Diagnostic and Statistical Manual of Mental Disorders, Fifth Edition
TP: Total protein
BUN: Blood urea nitrogen
AST: Aspartate aminotransferase
ALT: Alanine aminotransferase
LDH: Lactate dehydrogenase
ALP: Alkaline phosphatase
GGT: Gamma glutamyl transferase
WBC: White blood cells
RBC: Red blood cells

## References

[1] Treasure J, Duarte TA, Schmidt U. Eating disorders. Lancet. 2020;395(10227):899–911. 32171414 10.1016/S0140-6736(20)30059-3

[2] Mitchell JE, Peterson CB. Anorexia nervosa. N Engl J Med. 2020;382(14):1343–51. 32242359 10.1056/NEJMcp1803175

[3] Nielsen S, Moller-Madsen S, Isager T, Jorgensen J, Pagsberg K, Theander S. Standardized mortality in eating disorders–a quantitative summary of previously published and new evidence. J Psychosom Res. 1998;44(3–4):413–34. 9587884 10.1016/s0022-3999(97)00267-5

[4] Mehler PS, Brown C. Anorexia nervosa - medical complications. J Eat Disord. 2015;3:11. 25834735 10.1186/s40337-015-0040-8 PMC4381361

[5] Rebouche CJ. Kinetics, pharmacokinetics, and regulation of L-carnitine and acetyl-L-carnitine metabolism. Ann N Y Acad Sci. 2004;1033:30–41. 15591001 10.1196/annals.1320.003

[6] Breningstall GN. Carnitine deficiency syndromes. Pediatr Neurol. 1990;6(2):75–81. 2187442 10.1016/0887-8994(90)90037-2

[7] Longo N, Frigeni M, Pasquali M. Carnitine transport and fatty acid oxidation. Biochim Biophys Acta. 2016;1863(10):2422–35. 26828774 10.1016/j.bbamcr.2016.01.023 PMC4967041

[8] Hoppel C. The role of carnitine in normal and altered fatty acid metabolism. Am J Kidney Dis. 2003;41(4 Suppl 4):S4–12. 12751049 10.1016/s0272-6386(03)00112-4

[9] Flanagan JL, Simmons PA, Vehige J, Willcox MD, Garrett Q. Role of carnitine in disease. Nutr Metab (Lond). 2010;7:30. 20398344 10.1186/1743-7075-7-30 PMC2861661

[10] Pons R, De Vivo DC. Primary and secondary carnitine deficiency syndromes. J Child Neurol. 1995;10(Suppl 2):S8–24. 8576570

[11] Winter SC. Treatment of carnitine deficiency. J Inherit Metab Dis. 2003;26(2–3):171–80. 12889658 10.1023/a:1024433100257

[12] Imaeda M, Tanaka S, Fujishiro H, Kato S, Ishigami M, Kawano N, et al. Risk factors for elevated liver enzymes during refeeding of severely malnourished patients with eating disorders: a retrospective cohort study. J Eat Disord. 2016;4:37. 27980772 10.1186/s40337-016-0127-x PMC5142434

[13] Quigley EM, Marsh MN, Shaffer JL, Markin RS. Hepatobiliary complications of total parenteral nutrition. Gastroenterology. 1993;104(1):286–301. 8419252 10.1016/0016-5085(93)90864-9

[14] Fukusako T, Negoro K, Tsuda N, Kato M, Morimatsu M. [A case of secondary carnitine deficiency due to anorexia nervosa and severe liver damage]. Rinsho Shinkeigaku. 1995;35(1):34–7. 7781212

[15] Okamoto K, NT, Komaki G, Takii M, Kawai K, Matsumoto Y, Murakami S, Kubo C. A case of Anorexia Nervoxa with serum Carnitine Deficiency and High Creatine Phosphokinase Concentration during Nutritional Rehabilitation. JPM J Psychosom Med. 2001;41:369–75.

[16] Morton J, McLoughlin DM, Whiting S, Russell GF. Carnitine levels in patients with skeletal myopathy due to anorexia nervosa before and after refeeding. Int J Eat Disord. 1999;26(3):341–4. 10441250 10.1002/(sici)1098-108x(199911)26:3<341::aid-eat12>3.0.co;2-9

[17] Fotino AD, Sherma A. Patient with eating disorder, carnitine deficiency and dilated cardiomyopathy. J La State Med Soc. 2015;167(4):162–4. 27159507

[18] American Psychiatric Association. Diagnostic and statistical manual of mental disorders. 5th ed. American Psychiatric Publishing; 2013.

[19] American Psychiatric Association. Practice guideline for the treament of patients with eating disorders. 4th ed. American Psychiatric Publishing; 2023. 10.1176/appi.ajp.23180001 36722117

[20] Takahashi M, Ueda S, Misaki H, Sugiyama N, Matsumoto K, Matsuo N, et al. Carnitine determination by an enzymatic cycling method with carnitine dehydrogenase. Clin Chem. 1994;40(5):817–21. 8174257

[21] Japan Pediatric Society. Diagnostic and therapeutic guidelines for carnitine deficiency 2018: Japan Pediatric Society. 2018. In Japanese. https://www.jpeds.or.jp/uploads/files/20181207_shishin.pdf. Accessed 28 Jan 2024.

[22] Gopal DV, Rosen HR. Abnormal findings on liver function tests. Interpreting results to narrow the diagnosis and establish a prognosis. Postgrad Med. 2000;107(2):100. 5–9, 13 – 4. 10689411 10.3810/pgm.2000.02.869

[23] Kawai K, Yamashita S, Yamanaka T, Gondo M, Morita C, Nozaki T, et al. The longitudinal BMI pattern and body composition of patients with anorexia nervosa who require urgent hospitalization: a case control study. Biopsychosoc Med. 2011;5:14. 22142436 10.1186/1751-0759-5-14 PMC3275451

[24] Golden NH, Meyer W. Nutritional rehabilitation of anorexia nervosa. Goals and dangers. Int J Adolesc Med Health. 2004;16(2):131–44. 15266992 10.1515/ijamh.2004.16.2.131

[25] Labonia WD. L-carnitine effects on anemia in hemodialyzed patients treated with erythropoietin. Am J Kidney Dis. 1995;26(5):757–64. 7485128 10.1016/0272-6386(95)90439-5

[26] Arduini A, Rossi M, Mancinelli G, Belfiglio M, Scurti R, Radatti G, et al. Effect of L-carnitine and acetyl-L-carnitine on the human erythrocyte membrane stability and deformability. Life Sci. 1990;47(26):2395–400. 2263166 10.1016/0024-3205(90)90483-8

[27] Arduini A, Bonomini M, Clutterbuck EJ, Laffan MA, Pusey CD. Effect of L-carnitine administration on erythrocyte survival in haemodialysis patients. Nephrol Dial Transpl. 2006;21(9):2671–2. 10.1093/ndt/gfl155 16611680

[28] Hurot JM, Cucherat M, Haugh M, Fouque D. Effects of L-carnitine supplementation in maintenance hemodialysis patients: a systematic review. J Am Soc Nephrol. 2002;13(3):708–14. 11856775 10.1681/ASN.V133708

[29] Macher AD, Palazuelos D, Maviglia SM. Fatty emaciation: a case report of suspected fat overload syndrome in oral refeeding. JPEN J Parenter Enter Nutr. 2012;36(4):481–4. 10.1177/0148607111430227 22301330

[30] Khan L, Bamji MS. Plasma carnitine levels in children with protein-calorie malnutrition before and after rehabilitation. Clin Chim Acta. 1977;75(1):163–6. 403033 10.1016/0009-8981(77)90513-7

[31] Alp H, Orbak Z, Akcay F, Tan H, Aksoy H. Plasma and urine carnitine levels and carnitine supplementation in children with malnutrition. J Trop Pediatr. 1999;45(5):294–6. 10584472 10.1093/tropej/45.5.294

[32] Iwamoto J, Honda A, Miyamoto Y, Miyazaki T, Murakami M, Saito Y, et al. Serum carnitine as an independent biomarker of malnutrition in patients with impaired oral intake. J Clin Biochem Nutr. 2014;55(3):221–7. 25411530 10.3164/jcbn.14-77 PMC4227833

[33] Sharp CW, Freeman CP. The medical complications of anorexia nervosa. Br J Psychiatry. 1993;162:452–62. 8481735 10.1192/bjp.162.4.452

[34] Dowman J, Arulraj R, Chesner I. Recurrent acute hepatic dysfunction in severe anorexia nervosa. Int J Eat Disord. 2010;43(8):770–2. 19950111 10.1002/eat.20775

[35] Tomita K, Haga H, Ishii G, Katsumi T, Sato C, Aso R et al. Clinical manifestations of liver injury in patients with anorexia nervosa. Hepatol Res. 2013. 10.1111/hepr.12202 23841576

[36] Rautou PE, Cazals-Hatem D, Moreau R, Francoz C, Feldmann G, Lebrec D, et al. Acute liver cell damage in patients with anorexia nervosa: a possible role of starvation-induced hepatocyte autophagy. Gastroenterology. 2008;135(3):840–8. 8 e1-3. 18644371 10.1053/j.gastro.2008.05.055

[37] Restellini S, Spahr L, Rubbia Brandt L. Severe starvation-induced hepatocyte autophagy as a cause of acute liver injury in anorexia nervosa: a case report. Case Rep Hepatol. 2013;2013:749169. 10.1155/2013/749169 PMC4208431 25379300

[38] Ozawa Y, Shimizu T, Shishiba Y. Elevation of serum aminotransferase as a sign of multiorgan-disorders in severely emaciated anorexia nervosa. Intern Med. 1998;37(1):32–9. 9510397 10.2169/internalmedicine.37.32

[39] Tsukamoto M, Tanaka A, Arai M, Ishii N, Ohta D, Horiki N, et al. Hepatocellular injuries observed in patients with an eating disorder prior to nutritional treatment. Intern Med. 2008;47(16):1447–50. 18703853 10.2169/internalmedicine.47.0824

[40] Hanachi M, Melchior JC, Crenn P. Hypertransaminasemia in severely malnourished adult anorexia nervosa patients: risk factors and evolution under enteral nutrition. Clin Nutr. 2013;32(3):391–5. 22986227 10.1016/j.clnu.2012.08.020

[41] Nagata JM, Park KT, Colditz K, Golden NH. Associations of elevated liver enzymes among hospitalized adolescents with anorexia nervosa. J Pediatr. 2015;166(2):439–43. e1. 25477162 10.1016/j.jpeds.2014.10.048

[42] Rosen E, Sabel AL, Brinton JT, Catanach B, Gaudiani JL, Mehler PS. Liver dysfunction in patients with severe anorexia nervosa. Int J Eat Disord. 2016;49(2):151–8. 26346046 10.1002/eat.22436

[43] Karlic H, Schuster D, Varga F, Klindert G, Lapin A, Haslberger A, et al. Vegetarian diet affects genes of oxidative metabolism and collagen synthesis. Ann Nutr Metab. 2008;53(1):29–32. 18772587 10.1159/000152871

[44] Paulson DJ. Carnitine deficiency-induced cardiomyopathy. Mol Cell Biochem. 1998;180(1–2):33–4. 9546628

