# Correction: Secondary carnitine deficiency during refeeding in severely malnourished patients with eating disorders: a retrospective cohort study

**DOI:** 10.1186/s40337-026-01567-0

**Published:** 2026-03-18

**Authors:** Miho Imaeda, Satoshi Tanaka, Tomoko Oya-Ito, Mariko Uematsu, Hidetsugu Fujigaki, Kuniaki Saito, Masahiko Ando, Norio Ozaki

**Affiliations:** 1 Department of Clinical Oncology and Chemotherapy, Nagoya University Hospital, 65 Tsurumai, Showa, Nagoya, 466-8550 Aichi Japan; 2 Department of Psychiatry, Nagoya University Graduate School of Medicine, 65 Tsurumai, Showa, Nagoya, 466-8550 Aichi Japan; 3 NHO Higashiowari National Hospital, 1301, Omorikita 2, Moriyama, Nagoya, 463-0802 Aichi Japan; 4 NHO Nagoya Medical Center, 1-1, Sannomaru 4, Naka, Nagoya, 460-0001 Aichi Japan; 5 Department of Nutrition, Shubun University, 6 Nikkocho, Ichinomiya, 491-0938 Aichi Japan; 6 Department of Advanced Diagnostic System Development, Fujita Health University Graduate School of Health Sciences, 1-98 dengakugakubo, kutukakecho, Toyoake, 470-1192 Aichi Japan; 7 Center for Advanced Medicine and Clinical Research, Nagoya University Hospital, 65 Tsurumai, Showa, Nagoya, 466-8550 Aichi Japan; 8 Pathophysiology of Mental Disorders, Nagoya University Graduate School of Medicine, Institute for Glyco-core Research (iGCORE), Nagoya University, 65 Tsurumai, Showa, Nagoya, 466-8550 Aichi Japan

**Correction: Journal of Eating Disorders (2024) 12:97** 10.1186/s40337-024-01054-4

Following the publication of the original article, the author reported that in Table 1, the position of ‘Patients (N = 56)’ was incorrect. It should appear above ‘Median’, as shown below.

**Table 1:** Comparison between the patient groups and controls

	Controls (*N* = 35)		Patients (*N*=56)			
	Median (IQR)	Range	Median (IQR)	Range	*p* value^a^	*r*
Age (years)	24 (21–39)	20–49	26 (21–35)	17–53	0.7655	0.03
Height (m)	1.58 (1.546–1.611)	1.43–1.73	1.559 (1.528–1.597)	1.425–1.67	0.0976	0.17
Weight (kg)	49.5 (46.6–59)	42.2–85	30.1 (26.6–34.8)	18.9–44.5	< 0.0001	0.83
BMI at time of carnitine measurement (kg/m^2^)	20 (18.8–22.6)	16.6–34.2	13.8 (12.8–14.8)	10.8–18	< 0.0001	0.83
Free carnitine (36–74 µmol/L)	37.8 (34.7–40.5)	13.3–56.8	32.0 (23.2–41.5)	12.7–61.6	0.0249	0.24
Acylcarnitine (6–23 µmol/L)	7 (5.7–9.1)	3.0–11.3	3.8 (2.5–5.8)	0.7–9.9	< 0.0001	0.59
Total carnitine (45–91 µmol/L)	44.7 (41–48.9)	20.6–67.6	36.3 (26.1–46.2)	13.4–71.1	0.0012	0.34
Acylcarnitine/free carnitine (AC/FC)	0.19 (0.16–0.24)	0.07–0.55	0.12 (0.1–0.16)	0.04–0.27	< 0.0001	0.57

From: Secondary carnitine deficiency during refeeding in severely malnourished patients with eating disorders: a retrospective cohort study

*IQR* interquartile range,* BMI* body mass index

^a^Mann–Whitney U test

The original article (Imaeda et al. 2024) has been corrected.
